# The first documentation of the Nearctic–Paleotropical migratory route of the Arctic Warbler

**DOI:** 10.1002/ece3.9223

**Published:** 2022-09-09

**Authors:** Evan M. Adams, Iain J. Stenhouse, Andrew T. Gilbert, Jill Boelsma, George Gress, C. Scott Weidensaul, Charles Grigsby, Emily J. Williams, Laura Phillips, Carol L. McIntyre

**Affiliations:** ^1^ Biodiversity Research Institute Portland Maine USA; ^2^ Denali Education Center, Denali National Park McKinley Park Alaska USA; ^3^ Self‐employed Reinholds Pennsylvania USA; ^4^ Self‐employed Milton New Hampshire USA; ^5^ Denali National Park and Preserve Fairbanks Alaska USA

**Keywords:** Arctic Warbler, bird migration, light‐level data logger, Nearctic–Paleotropic migration, non‐breeding grounds

## Abstract

The Arctic Warbler (*Phylloscopus borealis*) is a cryptically plumed songbird with an uncommon Nearctic–Paleotropical migratory strategy. Using light‐level geolocators, we provide the first documentation of the migratory routes and wintering locations of two territorial adult male Arctic Warblers from Denali National Park and Preserve, Alaska. After accounting for position estimation uncertainties and biases, we found that both individuals departed their breeding grounds in early September, stopped over in southeastern Russia and China during autumn migration, then wintered in the Philippines and the island of Palau. Our documentation of Arctic Warbler wintering on Palau suggests that additional study is needed to document their wintering range. Our study provides hitherto unknown information on stopover and wintering locations for Arctic Warblers and indicates that this species may migrate further overwater than previously thought.

## Graphical abstract

The Arctic Warbler is a Nearctic–Paleotropical migrant—a rare strategy for songbirds—and a species of conservation concern that presents few analogs for comparison. This study presents the first individual tracks of migratory routes for the species using light‐logging data recorders. Using data from two individuals, we describe the timing of migration, high‐use areas during migration, and discover a possible new wintering location for the species.

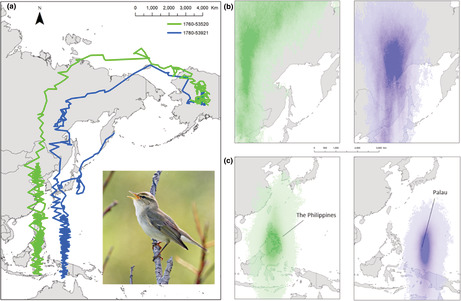

## 1 INTRODUCTION

Description of broadscale migratory movements in songbirds has begun using individual tracking (McKinnon & Love, 2018), but many species and migratory routes lack representation in the current state of the science. The Arctic Warbler (*Phylloscopus borealis*) is one of a handful of songbirds that make an annual journey from the Old World to the New World (Bairlein et al., 2012; Kessel, 1989) and is the only species in the genus *Phylloscopus* that breeds in North America (Bairlein et al., 2006). Despite recent research detecting cryptic species in the clade (Alstrӧm et al., 2011), information on Arctic Warbler breeding and movement ecology in North America is scarce (Kessel, 1989; Lowther & Sharbough, 2020). Alaskan migratory strategies are bifurcated around the 150th meridian, with western Alaskan birds often taking western migratory routes (Sivakumar et al., 2021); Arctic Warblers fall into this group as they fly a southwestern route to wintering grounds in Southeast Asia. Little is known about their passage in either geography or timing (Alerstam et al., 2008; Kessel, 1989; Lowther & Sharbough, 2020), despite the route providing a mechanism for the transcontinental spread of avian influenza (Winker et al., 2007) and Arctic Warblers being a high‐priority study species (Pearce & Ramey, 2006).

The Arctic Warbler's conservation status is currently unclear, although it is considered a species of continental stewardship in Alaska (Handel et al., 2021). Like nearly all migratory songbirds that nest in Alaska, the Arctic Warbler spends only a fraction of its annual cycle at the breeding grounds (approximately June through August). Moreover, movements and connectivity across the full annual cycle can be a critical driver of speciation and further complicate conservation activities (Webster & Marra, 2005). Thus, effective conservation efforts require knowledge of space and resource use during the breeding and non‐breeding season and an understanding of migratory movement and connectivity (Runge et al., 2014). Using light‐level geolocators to track individual movements (Cooper et al., 2017; Deluca et al., 2015; Jahn et al., 2013; McKinnon & Love, 2018; Tonra et al., 2019), we describe results from the first individual tracking study of Arctic Warblers, including migration behavior, migratory routes, and wintering locations of two individuals.

## 2 METHODS

Arctic Warblers were captured on their breeding grounds where we affixed light‐level geolocators to males. After tagged birds were recaptured the following year and the data were downloaded from the data logger, we used Bayesian movement models to determine the most likely daily positions of these animals and estimate migratory routes and timings.

### 2.1 Geolocator deployment and recovery

From 2016 to 2019, we captured adult male Arctic Warblers using mist nets with decoys and playback of conspecific songs and alarm calls. We banded each bird with a USGS aluminum leg band, sexed and aged them (Pyle, 1997), measured their mass with a digital scale (±0.01 g), and took standard morphological measurements. We also collected incidentally shed feathers in 2017 and feather and blood samples from 10 birds for contaminant analysis in 2018 (Stenhouse et al., 2019). In 2016, we attached 0.4 g archival light‐level geolocators (Lotek ML6040) to 27 after‐second‐year (adult) males. In 2018, we attached 0.3 g geolocators (Intigeo tags; Migrate Tech) to 15 adult males. We attached geolocators to birds in both years using a modified Rappole–Tipton harness (Rappole & Tipton, 1991; Streby et al., 2015) constructed of 0.5 mm black stretch jewelry cord. We assessed and adjusted the harness fit before release. Geolocator mass was approximately 3%–4% of the body mass of each bird. All geolocators were deployed on presumed breeding territories in Denali National Park and Preserve, Alaska (63.49° N, 150.07° W), from early June to early July, with most (33 of 42) deployed within a 101‐ha study area.

Initially, we attempted to recapture tagged birds using the same capture methods at the initial capture sites from early June to early July in subsequent years (2017–2019). When tagged birds were not sighted or recaptured at these sites, we expanded our recapture efforts to suitable breeding habitats (i.e., tall shrubs in riparian zones) within 500 m of the initial capture sites. Upon recapturing tagged birds, we recorded their band number, removed the geolocator, measured the bird, and released it. The data were then downloaded and readied for processing.

### 2.2 Geolocator data processing

After downloading the data from the recovered geolocators, twilight times were estimated using the BAStag package in R (R Core Team, 2022; Wotherspoon et al., 2016). A light threshold of 1.5 with a dark time minimum of 30 min was used to calculate initial estimates of twilight. A biologist audited this process's results and edited twilights when they were obviously incorrect based on the surrounding estimates. The intervention was rare (e.g., no more than three edits were made per track) to avoid process reproducibility issues. Latitude estimation is unreliable around the equinoxes and we only used values 2 weeks or more away from these days. These twilight times were used to estimate initial positions for each day. Outlier positions with more than 2 standard deviations from the running 7‐day average of twilight times were removed (Lisovski et al., 2016).

### 2.3 Movement analysis

We estimated the zenith angle of the filtered data over the wintering period using the Hill–Ekstrom method to calibrate each tag for differences in light detection (package probGLS; Lisovski et al., 2012). Similar processes for estimating zenith angle at the breeding grounds using known capture locations were challenging due to limited nighttime and transmitters being attached later in the breeding season. We assumed each individual was stationary from November 15 to April 10 to use the winter calibration method. Other calibration techniques were tested, although we found only slight variation in the predicted tracks based on the different calibrations. A threshold model estimated latitude and longitude after correcting for twilight bias during the calibration period. Data collection began too late in the summer for one Arctic Warbler (USGS band 1780‐53921) to effectively calibrate the geolocator, so we used calibration data from the other Arctic Warbler (1760‐53520) as the best estimate of twilight detection bias for that individual.

The Markov Chain Monte Carlo (MCMC) modeling process used to estimate position was implemented using the SGAT package in R (Lisovski & Hahn, 2012; R Core Team, 2022; Sumner et al., 2009). These models combine a position estimation model with a movement model to determine the animal's path in a Bayesian framework. We used estimates of passerine migratory movements to parameterize the movement speed and bearing parameters of the movement model. The distribution of terrestrial habitat contributed to the posterior estimates of position. We created a raster that categorized each cell as land or water and built a probability mask where overland travel was given a higher prior probability of use [log(2) vs. log(1), for example] (Hill & Renfrew, 2019). However, position estimation was unrestricted across the globe. Thus, while the probability mask increases the likelihood of terrestrial position estimates, it does not prevent overwater position estimates. Three MCMC chains were run with a 5000 iteration burn‐in, then a 10,000‐iteration posterior sample. Posterior estimates of locations were visually checked for chain convergence.

Final location estimates are the median of the three‐chain posterior for each position, and the uncertainty in each is displayed using the point intensity of the location estimate MCMC posteriors across a grid system. We estimated arrival and departure dates from the breeding and wintering grounds using multiple changepoint analyses to detect shifts in modeled latitude with a segmented neighborhood method (Killick & Eckley, 2014). We used a cumulative probability test for non‐normal data, with a penalty value of 0.9 to identify changes in mean longitude (for departing the breeding grounds) and latitude (for arriving at the wintering grounds). Estimated transition dates were visually assessed to determine accuracy, and data are publically available on Movebank.

## 3 RESULTS AND DISCUSSION

**FIGURE 1:**
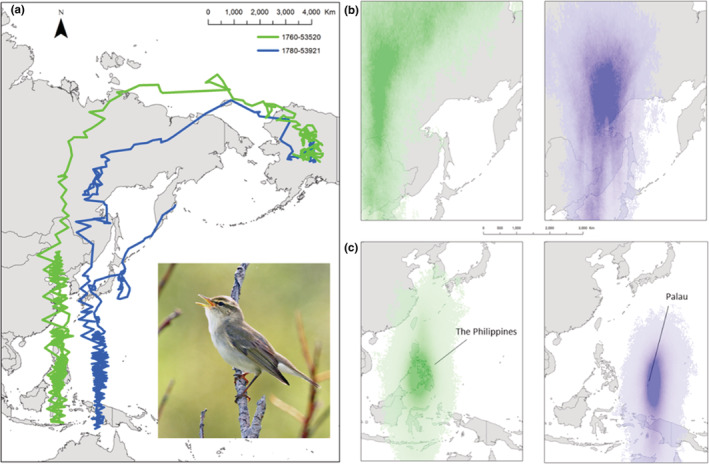
Median location estimates for two adult male Arctic Warblers from summer to spring (a). Space use maps describing the position estimation posterior distribution from the Bayesian state‐space model during fall migration (top: b, c) and winter (bottom: b, c). The color of the line and the space use maps are the same among individuals (green = 1760‐53520 tagged in 2016 and blue = 1780‐52921 tagged in 2018). Step length and bearing were estimated for each individual, and a movement model was used to improve location estimates from these light‐logging geolocators. Photo: Alan Schmierer (CC 1.0).

We recaptured 6 of the 61 adult male Arctic Warblers (9.8%) banded from 2016 to 2018, including 4 of the 42 (9.5%) with attached geolocators. All recaptures occurred within the 101‐ha study area and we had no evidence that geolocators fell off (Appendix S1). The distance between capture and recapture locations in subsequent years for the four tagged birds averaged 213 m (range = 120–365 m). Two of the four geolocators had useful data to track individuals from their breeding territories to their wintering grounds in Southeast Asia, one of which also extended into the early portion of spring migration (Figure 1). Unfortunately, the other two tags failed.

**FIGURE 2:**
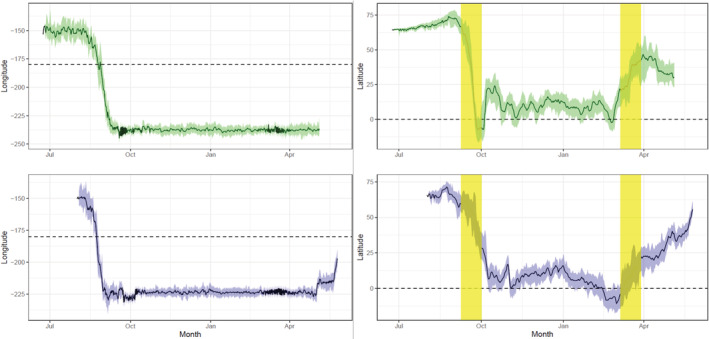
Median latitude and longitude estimates for each individual with 95% credible intervals (green = 1760‐53520 tagged in 2016 and blue = 1780‐52921 tagged in 2018). Yellow areas in the latitude figures indicate periods of time where latitude was not estimable using the raw data and position estimates are only based on model inputs. Dashed lines represent the international date line and the equator, note that longitude values west of the dateline do not reset to positive values.

The warblers departed the breeding grounds in Denali by 8/29/18 and 9/3/16 (1780‐53921 and 1760‐53520, respectively) and flew northwest, leaving North America from the western edge of the Lisburne Peninsula (Figure 1). They arrived at the wintering grounds by 10/20 and 10/15, respectively (Figure 2). The autumn migration route included overwater flights from Alaska to Russia and China and Japan into the Philippines and the surrounding Pacific Islands (Figure 1a). Both individuals appeared to linger in southeastern Russia, then again in southeast China and Japan, during fall migration before arriving at their respective wintering grounds (Figure 1b). Their autumn migration orientation is apparently consistent with the great circle migratory pattern reported by Alerstam et al. (2008), although more information is needed to confirm this observation. The birds traveled approximately 8500 and 8700 km to the mean wintering locations and averaged 197 and 181 km/day, respectively. One individual's wintering location was centered on the island of Palawan in the Philippines, while the other individual's wintering location was centered on Palau (Figure 1c). There is no prior evidence of Arctic Warblers wintering on Palau, either in the eBird database or in the English scientific literature (Dendy et al., 2015; Olsen & Eberdong, 2009; VanderWerf et al., 2006). Both geolocators failed before the birds returned to Alaska, but a portion of spring migration along the western coast of Japan and the Kamchatka Peninsula was documented for one bird (1780‐53921).

All previous evidence suggested that Arctic Warblers primarily wintered in the Philippines (Lowther & Sharbough, 2020), necessitating overwater migration to their breeding grounds. However, wintering on remote islands like Palau (about 890 km from the Philippines) requires more and/or longer overwater flights. Such flights are not uncommon for small birds; many songbirds and hummingbirds make a flight of similar length across the Gulf of Mexico annually (Deppe et al., 2015; Weidensaul et al., 2020). However, extending such flights increase mortality risk during migration due to encounters with extreme weather events (Newton, 2007). Given the potential for longer overwater flights, the importance of stopover habitat before those legs increases as greater fuel loads are required for successful flights (Vincze et al., 2019).

The geolocators we used in our study are low‐precision position estimation tools that can be persistently shaded by feathers and vegetation. Bird behavior and environmental conditions can cause errors in the data collection that systematically bias the results (Fudickar et al., 2012). Thus, these position estimates could be confounded by systemic shading issues and the different tag models for each individual (Intigeo for 1780‐53921 and Lotek for 1760‐53520). Therefore, our confidence in the Palau wintering location is not high, given the lack of prior evidence of this species wintering there. While our data suggest that one bird wintered on Palau, this pattern could be influenced by a systematic bias in sunrise or sunset time estimation. These biases can be quantified in some situations, but both data loggers stopped recording before recapturing. Alternatively, if these data are correct, we have documented a previously unknown wintering location for the species, and the winter range for this species should be reevaluated. Given the lack of eBird data collected on the island, the non‐descript plumage of *Phylloscopus* warblers, and the potential for recent range shifts, this overwinter location seems possible. While we found no evidence of our data being systematically biased, the result's novelty suggests that caution is needed until the Arctic Warbler's presence can be confirmed on Palau. More research and monitoring are needed for this species on the non‐breeding grounds; a range‐wide survey with expansion to potential locations that currently have poor survey effort is important to better understand its ecology.

This study has documented the migratory pathway and suggests new wintering locations for Arctic Warblers that breed in interior Alaska. Geolocators and similar methods that track movements across the full annual cycle in small songbirds continue to be powerful tools in illuminating idiosyncratic movements for poorly studied species and advancing our understanding of factors affecting the conservation of migrants with complex life histories (Tonra et al., 2019). However, the costs of such studies to the tagged individuals also need to be considered as does the probability of recapturing the tagged bird. Our recovery rate of tagged birds was 9.5%, much lower than the 35% resighting rate of color‐banded Arctic Warblers at a different study site, 210 km east of our study area (Lowther & Sharbough, 2020). Low recapture rates may have resulted from low breeding site fidelity and low survival rates (DeSante et al., 2015; Ralph et al., 1993), geolocators reducing survival (Brlík et al., 2020; Costantini & Møller, 2013), low detectability of tagged birds in the study area, low territoriality of males in the study area, or individual dispersal to new breeding habitat (Hedlund et al., 2017; Mizel et al., 2016). Furthermore, we noted intra‐season movements that indicated large territories or inter‐territory movements (average = 235 m, range = 85–630; *n* = 6 banded/tagged birds) that may have contributed to our low recapture rate. More information is needed on Arctic Warblers throughout their annual cycle, including their fidelity to breeding sites, to assess their conservation status (Runge et al., 2014) and maximize the return on studies such as this.

Arctic Warblers are currently altering their breeding range and habitat preference as a result of climate change at their Alaskan breeding grounds (Mizel et al., 2016; Thompson et al., 2016). Thus, this species' risk profile has increased, and we recommend expanding current efforts to understand Arctic Warbler population status and the ecological drivers of population trends across their annual cycle.

## AUTHOR CONTRIBUTIONS

**Evan M. Adams:** Data curation (lead); formal analysis (lead); investigation (lead); methodology (lead); validation (lead); visualization (lead); writing – original draft (lead); writing – review and editing (lead). **Iain J. Stenhouse:** Conceptualization (equal); investigation (equal); methodology (equal); visualization (supporting); writing – original draft (supporting); writing – review and editing (supporting). **Andrew T. Gilbert:** Methodology (supporting); validation (supporting); visualization (supporting); writing – original draft (supporting); writing – review and editing (supporting). **Jill Boelsma:** Investigation (supporting); methodology (supporting). **George Gress:** Investigation (supporting); methodology (supporting). **C. Scott Weidensaul:** Conceptualization (supporting); investigation (equal); methodology (equal); writing – original draft (supporting); writing – review and editing (supporting). **Charles Grigsby:** Investigation (supporting); methodology (supporting). **Emily J. Williams:** Investigation (equal); methodology (equal); project administration (supporting); supervision (supporting); writing – original draft (supporting); writing – review and editing (supporting). **Laura Phillips:** Investigation (equal); methodology (equal); project administration (supporting); supervision (supporting); writing – original draft (supporting); writing – review and editing (supporting). **Carol L. McIntyre:** Conceptualization (lead); data curation (supporting); funding acquisition (lead); investigation (equal); methodology (equal); project administration (lead); supervision (lead); visualization (supporting); writing – original draft (supporting); writing – review and editing (supporting).

## CONFLICTS OF INTEREST

The authors declare no conflicts of interest.

### OPEN RESEARCH BADGES

Is the author interested in applying for an Open Research Badge?: Yes. Our analysis code is accessible at: https://github.com/evanmadams/denali_songbird_tracking.

## Supporting information


Appendix S1

## Data Availability

Geolocator raw data are publically available on Movebank (www.movebank.org) at the Denali National Park Critical Connections project page https://www.movebank.org/cms/webapp?gwt_fragment=page%3Dstudies%2Cpath%3Dstudy2225554945.

## References

[ece39223-bib-0001] Alerstam, T. , Bäckman, J. , Strandberg, R. , Gudmundsson, G. A. , Hedenström, A. , Henningsson, S. S. , Karlsson, H. , & Rosén, M. (2008). Great‐circle migration of arctic passerines. The Auk, 125(4), 831–838. 10.1525/auk.2008.07142

[ece39223-bib-0002] Alstrӧm, P. , Saitoh, T. , Williams, D. , Nishiumi, I. , Shigeta, Y. , Ueda, F. , Irestedt, M. , Bjӧrklund, M. , & Olsson, U. (2011). The Arctic warbler *Phylloscopus borealis*–Three anciently separated cryptic species revealed. Ibis, 153(2), 395–410. 10.1111/j.1474-919X.2011.01116.x

[ece39223-bib-0003] Bairlein, F. , Alstrӧm, P. , Aymi, R. , Clement, P. , Dyrcz, A. , Gargallo, G. , Hawkins, F. , Madge, S. , Pearson, D. , & Svensson, L. (2006). Family Sylviidae (Warblers). In J. del Hoyo , A. Elliott , & D. A. Christie (Eds.), Handbook of the birds of the world (Vol. 11, pp. 492–709). Lynx Edici.

[ece39223-bib-0004] Bairlein, F. , Norris, D. R. , Nagel, R. , Bulte, M. , Voigt, C. C. , Fox, J. W. , Hussell, D. J. T. , & Schmaljohann, H. (2012). Cross‐hemisphere migration of a 25 g songbird. Biology Letters, 8(4), 505–507. 10.1098/rsbl.2011.1223 22337504 PMC3391447

[ece39223-bib-0005] Brlík, V. , Koleček, J. , Burgess, M. , Hahn, S. , Humple, D. , Krist, M. , Ouwehand, J. , Weiser, E. L. , Adamík, P. , Alves, J. A. , Arlt, D. , Barišić, S. , Becker, D. , Belda, E. J. , Beran, V. , Both, C. , Bravo, S. P. , Briedis, M. , Chutný, B. , … Procházka, P. (2020). Weak effects of geolocators on small birds: A meta‐analysis controlled for phylogeny and publication bias. Journal of Animal Ecology, 89(1), 207–220. 10.1111/1365-2656.12962 30771254

[ece39223-bib-0006] Cooper, N. W. , Hallworth, M. T. , & Marra, P. P. (2017). Light‐level geolocation reveals wintering distribution, migration routes, and primary stopover locations of an endangered long‐distance migratory songbird. Journal of Avian Biology, 48(2), 209–219. 10.1111/jav.01096

[ece39223-bib-0007] Costantini, D. , & Møller, A. P. (2013). A meta‐analysis of the effects of geolocator application on birds. Current Zoology, 59(6), 697–706. 10.1093/czoolo/59.6.697

[ece39223-bib-0008] Deluca, W. V. , Rimmer, C. C. , Mcfarland, K. P. , Deluca, W. V. , Woodworth, B. K. , Rimmer, C. C. , Marra, P. P. , Taylor, P. D. , Mcfarland, K. P. , Mackenzie, S. A. , & Norris, D. R. (2015). Transoceanic migration by a 12 g songbird transoceanic migration by a 12 g songbird. Biology Letters, 11, 20141045. 25832815 10.1098/rsbl.2014.1045 PMC4424611

[ece39223-bib-0009] Dendy, J. , Cordell, S. , Giardina, C. P. , Hwang, B. , Polloi, E. , & Rengulbai, K. (2015). The role of remnant forest patches for habitat restoration in degraded areas of Palau. Restoration Ecology, 23(6), 872–881. 10.1111/rec.12268

[ece39223-bib-0010] Deppe, J. L. , Ward, M. P. , Bolus, R. T. , Diehl, R. H. , Celis‐Murillo, A. , Zenzal, T. J. , Moore, F. R. , Benson, T. J. , Smolinsky, J. A. , Schofield, L. N. , Enstrom, D. A. , Paxton, E. H. , Bohrer, G. , Beveroth, T. A. , Raim, A. , Obringer, R. L. , Delaney, D. , & Cochran, W. W. (2015). Fat, weather, and date affect migratory songbirds' departure decisions, routes, and time it takes to cross the Gulf of Mexico. Proceedings of the National Academy of Sciences of the United States of America, 112(46), E6331–E6338. 10.1073/pnas.1503381112 26578793 PMC4655507

[ece39223-bib-0011] DeSante, D. F. , Kaschube, D. R. , & Saracco, J. F. (2015). Vital rates of North American landbirds . www.VitalRatesOfNorthAmericanLandbirds.org

[ece39223-bib-0012] Fudickar, A. M. , Wikelski, M. , & Partecke, J. (2012). Tracking migratory songbirds: Accuracy of light‐level loggers (geolocators) in forest habitats. Methods in Ecology and Evolution, 3(1), 47–52. 10.1111/j.2041-210X.2011.00136.x

[ece39223-bib-0013] Handel, C. M. , Stenhouse, I. J. , & Matsuoka, S. M. (2021). Arctic plains and mountains. In C. M. Handel , I. J. Stenhouse , & S. M. Matsuoka (Eds.), Alaska landbird conservation plan (pp. 57–72). Boreal Partners in Flight.

[ece39223-bib-0014] Hedlund, J. S. U. , Sjösten, F. , Sokolovskis, K. , & Jakobsson, S. (2017). Point of no return – Absence of returning birds in the otherwise philopatric willow warbler *Phylloscopus trochilus* . Journal of Avian Biology, 48(3), 399–406. 10.1111/jav.00973

[ece39223-bib-0015] Hill, J. M. , & Renfrew, R. B. (2019). Migratory patterns and connectivity of two North American grassland bird species. Ecology and Evolution, 9(1), 680–692. 10.1002/ece3.4795 30680148 PMC6342103

[ece39223-bib-0016] Jahn, A. E. , Levey, D. J. , Cueto, V. R. , Ledezma, J. P. , Tuero, D. T. , Fox, J. W. , & Masson, D. (2013). Long‐distance bird migration within South America revealed by light‐level geolocators. The Auk, 130(2), 223–229. 10.1525/auk.2013.12077

[ece39223-bib-0017] Kessel, B. (1989). Birds of the Seward Penninsula, Alaska: Their biogeography, seasonality, and natural history. University of Alasaka Press.

[ece39223-bib-0018] Killick, R. , & Eckley, I. A. (2014). Changepoint: An R package for changepoint analysis. Journal of Statistical Software, 58(3), 1–19. 10.18637/jss.v058.i03

[ece39223-bib-0019] Lisovski, S. , & Hahn, S. (2012). GeoLight ‐ processing and analysing light‐based geolocator data in R. Methods in Ecology and Evolution, 3(6), 1055–1059. 10.1111/j.2041-210X.2012.00248.x

[ece39223-bib-0020] Lisovski, S. , Hewson, C. M. , Klaassen, R. H. G. , Korner‐Nievergelt, F. , Kristensen, M. W. , & Hahn, S. (2012). Geolocation by light: Accuracy and precision affected by environmental factors. Methods in Ecology and Evolution, 3(3), 603–612. 10.1111/j.2041-210X.2012.00185.x

[ece39223-bib-0021] Lisovski, S. , Wotherspoon, S. J. , & Sumner, M. D. (2016). TwGeos: Basic data processing for light‐level geolocation archival tags . R Package Version 0.1.2.

[ece39223-bib-0022] Lowther, P. E. , & Sharbough, S. (2020). Arctic warbler (*Phylloscopus borealis*), version 1.0. In S. M. Billerman (Ed.), Birds of the world. Cornell Lab of Ornithology. 10.2173/bow.arcwar1.01

[ece39223-bib-0023] McKinnon, E. A. , & Love, O. P. (2018). Ten years tracking the migrations of small landbirds: Lessons learned in the golden age of bio‐logging. Auk, 135(4), 834–856. 10.1642/AUK-17-202.1

[ece39223-bib-0024] Mizel, J. D. , Schmidt, J. H. , McIntyre, C. L. , & Roland, C. A. (2016). Rapidly shifting elevational distributions of passerine species parallel vegetation change in the subarctic. Ecosphere, 7(3), 1–15. 10.1002/ecs2.1264

[ece39223-bib-0025] Newton, I. (2007). Weather‐related mass‐mortality events in migrants. Ibis, 149(3), 453–467. 10.1111/j.1474-919X.2007.00704.x

[ece39223-bib-0026] Olsen, A. R. , & Eberdong, M. (2009). Species richness and other noteworthy observations at an important bird area in Palau. Micron, 41(1), 59–69.19766011

[ece39223-bib-0027] Pearce, J. , & Ramey, A. (2006). High priority species for avian influenza in Alaska . https://www.usgs.gov/centers/asc/science/high‐priority‐species‐avian‐influenza‐alaska?qt‐science_center_objects=0#qt‐science_center_objects

[ece39223-bib-0028] Pyle, P. (1997). Identification guide to North American birds: A compendium of information on identifying, ageing, and sexing” near‐passerines” and passerines in the hand. Slate Creek Press.

[ece39223-bib-0029] R Core Team . (2022). R: A language and environment for statistical computing. R Foundation for Statistical Computing. https://www.r‐project.org/

[ece39223-bib-0030] Ralph, C. J. , Geupel, G. R. , Pyle, P. , Martin, T. E. , & DeSante, D. F. (1993). Handbook of field methods for monitoring landbirds. Director, 144(1), 1–41. http://digitalcommons.unl.edu/cgi/viewcontent.cgi?article=1104&context=usdafsfacpub

[ece39223-bib-0031] Rappole, J. H. , & Tipton, A. R. (1991). New harness design for attachment of radio transmitters to small passerines (nuevo Diseño de Arnés Para Atar Transmisores a Passeriformes Pequeños). Journal of Field Ornithology, 62(3), 335–337. http://www.jstor.org/stable/20065798

[ece39223-bib-0032] Runge, C. A. , Martin, T. G. , Possingham, H. P. , Willis, S. G. , & Fuller, R. A. (2014). Conserving mobile species. Frontiers in Ecology and the Environment, 12(7), 395–402.

[ece39223-bib-0033] Sivakumar, A. H. , Sheldon, D. , Winner, K. , Burt, C. S. , & Horton, K. G. (2021). A weather surveillance radar view of Alaska avian migration. Proceedings of the Royal Society B, 288, 20210232. 33947241 10.1098/rspb.2021.0232 PMC8097201

[ece39223-bib-0034] Stenhouse, I. J. , Adams, E. M. , Phillips, L. M. , Weidensaul, S. , & McIntyre, C. L. (2019). A preliminary assessment of mercury in the feathers of migratory songbirds breeding in the North American subarctic. Ecotoxicology, 1–8. 10.1007/s10646-019-02105-2 31531800

[ece39223-bib-0035] Streby, H. M. , McAllister, T. L. , Peterson, S. M. , Kramer, G. R. , Lehman, J. A. , & Andersen, D. E. (2015). Minimizing marker mass and handling time when attaching radiotransmitters and geolocators to small songbirds. Condor, 117(2), 249–255. 10.1650/CONDOR-14-182.1

[ece39223-bib-0036] Sumner, M. D. , Wotherspoon, S. J. , & Hindell, M. A. (2009). Bayesian estimation of animal movement from archival and satellite tags. PLoS One, 4(10), 19–22. 10.1371/journal.pone.0007324 PMC2758548 19823684

[ece39223-bib-0037] Thompson, S. J. , Handel, C. M. , Richardson, R. M. , & McNew, L. B. (2016). When winners become losers: Predicted nonlinear responses of arctic birds to increasing woody vegetation. PLoS One, 11(11), 1–17. 10.1371/journal.pone.0164755 PMC5112980 27851768

[ece39223-bib-0038] Tonra, C. M. , Hallworth, M. T. , Boves, T. J. , Reese, J. , Bulluck, L. P. , Johnson, M. , Viverette, C. , Percy, K. , Ames, E. M. , Matthews, A. , Slevin, M. C. , Wilson, R. R. , & Johnson, E. I. (2019). Concentration of a widespread breeding population in a few critically important nonbreeding areas: Migratory connectivity in the Prothonotary Warbler. Condor, 121(2), duz019. 10.1093/condor/duz019

[ece39223-bib-0039] VanderWerf, E. , Wiles, G. J. , Marshall, A. P. , & Kneight, M. (2006). Observations of migrants and other birds in Palau, April–May 2005, including the first Micronesian record of a Richard's pipit. Micronesica, 39, 11–29. http://university.uog.edu/up/micronesica/abstracts_39/pdfs_39/VanderWerf‐Palau.pdf

[ece39223-bib-0040] Vincze, O. , Vágási, C. I. , Pap, P. L. , Palmer, C. , & Møller, A. P. (2019). Wing morphology, flight type and migration distance predict accumulated fuel load in birds. Journal of Experimental Biology, 222(1), 4–10. 10.1242/jeb.183517 30446537

[ece39223-bib-0041] Webster, M. S. , & Marra, P. P. (2005). The importance of understanding migratory connectivity and seasonal interations. In R. S. Greenberg & P. P. Marra (Eds.), Birds of two worlds, the ecology and evolution of migration (pp. 199–209). Johns Hopkins University Press.

[ece39223-bib-0042] Weidensaul, S. , Robinson, T. R. , Sargent, R. R. , Sargent, M. B. , & Zenzal, T. J. (2020). Ruby‐throated hummingbird (*Archilochus colubris*), version 1.0. In P. G. Rodewald (Ed.), Birds of the world. Cornell Lab of Ornithology. 10.2173/bow.rthhum.01

[ece39223-bib-0043] Winker, K. , McCracken, K. G. , Gibson, D. D. , Pruett, C. L. , Meier, R. , Huettmann, F. , Wege, M. , Kulikova, I. V. , Zhuravlev, Y. N. , Perdue, M. L. , Spackman, E. , Suarez, D. L. , & Swayne, D. E. (2007). Movements of birds and avian influenza from Asia into Alaska. Emerging Infectious Diseases, 13(4), 547–552. 10.3201/eid1304.061072 17553268 PMC2725966

[ece39223-bib-0044] Wotherspoon, S. J. , Sumner, M. D. , & Lisovski, S. (2016). BAStag: Basic data processing for light based geolocation archival tags . R package version 0.1. 3. GitHub Repository.

