# Supplementary material for: The first documentation of the Nearctic–Paleotropical migratory route of the Arctic Warbler
Source: Ecol Evol. 2022 Sep 9;12(9):e9223. doi: 10.1002/ece3.9223 (PMC9461342; doi:10.1002/ece3.9223)
Supplement: Supplementary file 1 — Appendix S1 [file ECE3-12-e9223-s001.docx]

**Supplemental materials**

*Appendix A: R Code for analysis of geolocator data*

[https://github.com/evanmadams/denali_songbird_tracking]

*Appendix B: Information on tagged Arctic Warblers During the Breeding Season*

Table 1. Recapture histories for adult male Arctic Warblers banded and tagged with geolocators in Denali National Park and Preserve, Alaska, 2016-2019. Geolocators were deployed in 2016 and 2018. Recapture efforts occurred in 2017, 2018, and 2019.

|  |  | **Capture Dates** | | | |
| --- | --- | --- | --- | --- | --- |
| **Bird ID** | **Deployment Year** | **Initial Capture** | **Recapture 1** | **Recapture #2.** | **Recapture #3** |
| 53520 | 2016 | 6/22/2016 | 6/9/2017 |  |  |
| 53542 | 2016 | 6/23/2016 | 7/12/2017 | 6/19/2018 | 7/1/2019 |
| 53562 | 2016 | 7/6/2016 | 6/23/2017 |  |  |
| 53921 | 2018 | 6/23/2017 | 7/4/2018 | 7/3/2019 |  |
